# Development of an Active Training Method for Belt Conveyor

**DOI:** 10.3390/ijerph19010437

**Published:** 2021-12-31

**Authors:** Dawid Szurgacz, Sergey Zhironkin, Jiří Pokorný, A. J. S. (Sam) Spearing, Stefan Vöth, Michal Cehlár, Izabela Kowalewska

**Affiliations:** 1 Center of Hydraulics DOH Ltd., 41-906 Bytom, Poland; dawidszurgacz@vp.pl; 2 Polska Grupa Górnicza S.A., ul. Powstańców 30, 40-039 Katowice, Poland; 3 Department of Trade and Marketing, Siberian Federal University, 79 Svobodny av., 660041 Krasnoyarsk, Russia; 4 Department of Open Pit Mining, T.F. Gorbachev Kuzbass State Technical University, 28 Vesennya st., 650000 Kemerovo, Russia; 5 School of Core Engineering Education, National Research Tomsk Polytechnic University, 30 Lenina st., 634050 Tomsk, Russia; 6 Faculty of Safety Engineering, VSB—Technical University of Ostrava, Lumírova 13/630, 700 30 Ostrava-Výškovice, Czech Republic; jiri.pokorny@vsb.cz; 7 School of Mines, China University of Mining and Technology, 1 Daxue Road, Tongshan District, Xuzhou 221116, China; sam.spearing@curtin.edu.au; 8 Technische Hochschule Georg Agricola (THGA), Westhoffstraβe 15, 44791 Bochum, Germany; stefan.voeth@thga.de; 9 Faculty of Mining, Ecology, Process Technologies and Geotechnology, Institute of Earth Sources, Technical University of Košice, Letná 9, 042 00 Košice, Slovakia; michal.cehlar@tuke.sk; 10 Faculty of Geoengineering, Mining and Geology, Wroclaw University of Science and Technology, Na Grobli 15, 50-421Wroclaw, Poland; izabela.kowalew@gmail.com

**Keywords:** occupational stress, work safety, human factor, active training method, underground mining

## Abstract

The global situation related to the COVID-19 pandemic has forced employers to find an adequate way to conduct training in order to ensure work safety. The underground mining industry is one of the industries which, due to its nature, was not able to switch to remote work. Conducting traditional training risked spreading the virus among workers. For this purpose, it was necessary to start a search for a form of training that would be safe and would not cause additional stress for employees. Research on the development of an active employee training method and testing of the method itself was conducted online. In order to develop a method of active training, one of the most important workstations was selected, which is the operation of the conveyor belt. The training method comprises four training modules. The modules cover questions related to the operation of the conveyor belt, emergencies, its assembly and disassembly, repair and maintenance. The developed issues also take into account questions concerning natural hazards and work safety. The entire training course lasts 10 days. Every day, an employee receives a set of eight questions sent to their email address, which they must answer before starting work. The article describes the methodology and implementation of the training.

## 1. Introduction

In world literature [1,2,3], the topic of utility of conveyor belts has been widely discussed. Their numerous advantages were emphasized, such as high reliability [4,5,6] and the ease of adapting the conveyor belt’s route to a terrain with rich topography [7,8,9]. Another group of advantages includes, among others, continuity of transport, simple structure and relatively low energy consumption [10,11,12]. For the conveyor belt to work properly, adequately trained people are needed. They must know how to operate a given conveyor belt, how to maintain and repair it, as well as how to properly assemble and disassemble it [13,14,15].

Despite much important research released on conveyor belts [16,17,18] as well as employee training [19,20,21], the idea of combining these two things has never arisen. Usually, full-time employees are only tested on safety rules, and no one checks their knowledge of the comprehensive operation of the conveyor belt used in their mine. In order to meet the needs of mining plants, the issues of the role and importance of conveyor belts were raised, as well as the question of related risks and the idea of creating an active employee training program that aims at reducing work routine and increasing the level of knowledge. Work competences are presented in four modules, which are closely related to the work performed when operating a conveyor belt in a hard coal mine.

A properly selected training cycle [22] is the key to minimizing employees’ stress [23,24,25]. The use of innovative solutions [26,27,28] to adopt an adequate form of training can significantly influence the awareness of employees that such a course updates their current knowledge. Based on the literature concerning work safety aspects in deep mining plants and the analysis of the current trends in the education and training of mining staff [29,30,31], an active 10-day training course has been proposed [32]. The course consists of eight questions for each day.

The COVID-19 [33,34,35] pandemic has caused problems related to providing an adequate level of training for employees in the mines. For the sake of ensuring occupational safety and maintaining the knowledge of occupational health and safety, a program for active training of employees operating conveyor belts was developed as part of international cooperation and was implemented in the Polish Mining Group (Katowice, Poland).

The paper contains results of the research related to the development of an active training course for employees on posts related to service, repair, maintenance, as well as assembly and disassembly of conveyor belts. The pandemic has forced employers to look for new solutions or develop new tools related to employee training. In order to ensure safety and appropriate knowledge at the workplace and to meet the conditions resulting from work safety regulations [36,37,38], an active training course was developed. In the mines belonging to the Polish Mining Group, a group of employees was selected to participate in research on the development of the course in question. Each of the selected employees received eight questions each day via an app. The training lasted 10 working days. They had to answer the questions they received before starting work. The developed form of training included issues related to conveyor belts. The training was also supplemented with issues related to natural hazards and the principles of occupational health and safety. Research results obtained from the developed training course allowed for the implementation of this solution in the mining company.

## 2. Materials and Methods

The study was conducted according to the guidelines of the Declaration of Helsinki and approved by the Mining Institute Review Board of T.F. Gorbachev Kuzbass State Technical University (Kuzbass, Russia) (Protocol Number 1101 from 29 November 2021).

The main problem in conducting the training and obtaining significant research results was the COVID-19 situation. In order to conduct reliable research, a group of employees working at the conveyor belt service post in Polish Mining Group Inc in KWK ROW Ruch Chwałowice was gathered. Employees volunteered for the training. After defining the main areas of competence of employees servicing conveyor belts (emergency conditions, conveyor belt operation, repair and maintenance, assembly and disassembly), the tests, divided into two stages, were started:

1. Stage I—preparation of four training modules on conveyor belts and conducting research on a smaller group of people in order to verify the accuracy of the developed training course;
2. Stage II—on the basis of the results obtained from the first stage, an online course was prepared covering 10 training days.

The course on active training of employees working in the position related to the operation of the conveyor belt was divided into four parts in order to check the level of knowledge of employees in each of them. The developed modules include issues related to the operation of the conveyor belt, emergency conditions, repair and maintenance, as well as assembly and disassembly. Only such a detailed division would comprehensively check the entirety of knowledge that an employee working with conveyor belts in a hard coal mine should possess. Moreover, this division made it possible to identify all strengths and weaknesses in the given area of knowledge. The questions included in the course have been developed based on the available literature.

The tests were carried out as follows: employees that volunteered received a paper test with a different number of questions, depending on the module. They used a pen to mark the answers they believed were correct. They had about 20 min for it. They were all multiple-choice questions, but with different forms of answers (yes/no/don’t know, as well as A/B/C/D). After an employee handed over the completed test, it was checked manually. The aim of stage I was to develop adequately formulated questions on each of the modules so that they could be answered in a concise and unambiguous way, choosing the best possible answer. This contributed to the preparation of a proper online test kit (stage II). The tests were also intended to check the level of knowledge the employees related to the operation of conveyor belts in a hard coal mine had and whether there was a real need to develop this type of course.

The newly developed training method was designed to verify the employee’s level of knowledge. The developed training method was intended for employees with appropriate work experience and qualified to operate the conveyor. The introduction of a new method of employee training is expected to bring improvements in workplace safety. Twenty employees participated in the research to develop the new method. The presented training method will be implemented in 20 underground coal mines. About 8000 employees will benefit from the developed form of training. Table 1 shows the questions and correct answers from the selected training day.

## 3. Results

The questions related to the operation of a conveyor belt in a hard coal mine focused on checking the elementary knowledge that an employee dealing with the operation of the said conveyor belt should have. The developed questions verified whether the respondent knew when they could safely use the conveyor belt and how to do it in order not to expose themselves or their colleagues to danger, as well as not to damage the mechanism of the conveyor belt itself. The issues related to emergency conditions belong to the most important module because many people’s lives may depend on knowing and following safety rules. Every employee should know how to behave in dangerous situations that threaten theirs or their co-workers’ health or life.

An employee working with a conveyor belt in a hard coal mine should be able to repair the machine and carry out its maintenance in order to extend its usefulness, as well as to increase its efficiency. The developed questions’ function was to verify whether the employee knows, among other things, how often a detailed inspection or maintenance should be carried out and what to do in this case. Efficient operation of the conveyor belt, the ability to repair and maintain it, as well as emergency conditions conduct are extremely important; however, it should be remembered that in order for a given employee to have comprehensive, full knowledge of how to operate the conveyor belt, they must also deal with its assembly and disassembly, because this has a significant impact on the further possibility of proper use of the conveyor belt.

**Figure 1:**
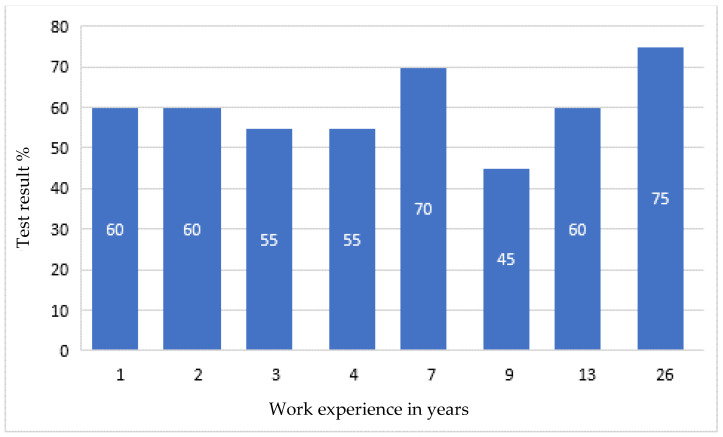
Research results regarding the operation of the conveyor belt, depending on professional experience.

**Figure 2:**
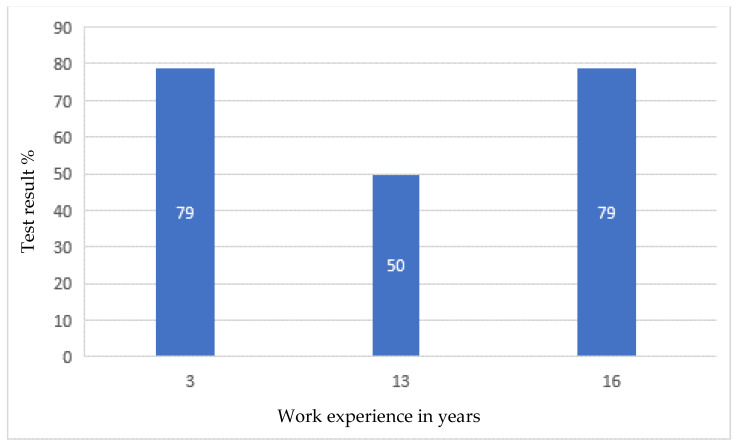
Research results regarding the emergencies of the conveyor belt, depending on professional experience.

**Figure 3:**
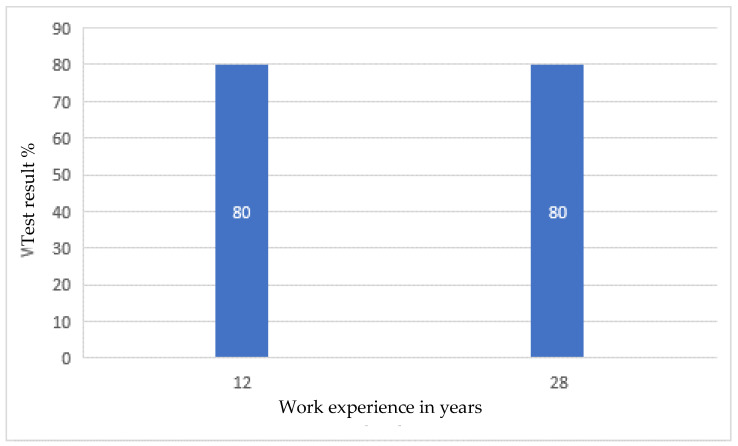
Research results regarding repair and maintenance of the conveyor belt, depending on professional experience.

**Figure 4:**
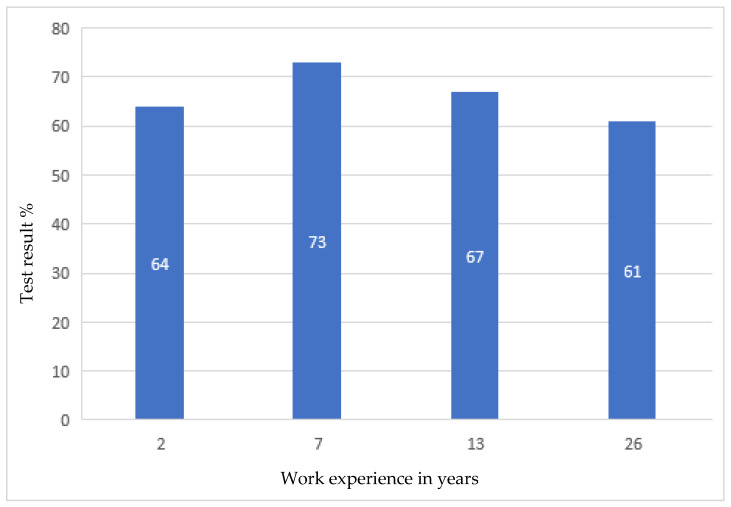
Research results regarding assembly and disassembly of the conveyor belt, depending on professional experience.

After the tests, none of the employees had any major comments as to the form and content of the questions. This suggested that, on the basis of the developed modules, it was possible to create an online version that employees would complete every day for 10 days. After verifying and checking the answers, it turned out that even the most senior employees did not answer all the questions correctly. Moreover, longer work experience did not lead to a higher result, as shown in Figure 1, Figure 2, Figure 3 and Figure 4.

Developing the questions in the modules and conducting the initial research was one of the most important parts of creating an active training course for employees who operate conveyor belts. Stage I allowed for final approval of the questions and made it possible to use them in the online course. The above results (Figure 1, Figure 2, Figure 3 and Figure 4) allowed us to validate the questions and enable their use in the online course. The results confirm that it is necessary to conduct more frequent training sessions among employees, as their knowledge turned out to be unsatisfactory.

## 4. Discussion

After the final form and content of the questions had been approved, the process of creating an electronic form of active training course for employees operating conveyor belts in an underground coal mine was started. A total of 10 quizzes over ten working days, or two training weeks, were created. Each of the quizzes contained eight randomly selected questions, so that each test had at least one question from the previously developed modules. The questions were not repeated. Each day, the employee received a link by email at any time of their choice. After clicking on the link, the employee displayed a window where they had to enter their email address and answer the questions. Each of the questions had the requirement of an answer, which resulted in the respondent not being able to return the quiz without answering every question.

**Figure 5:**
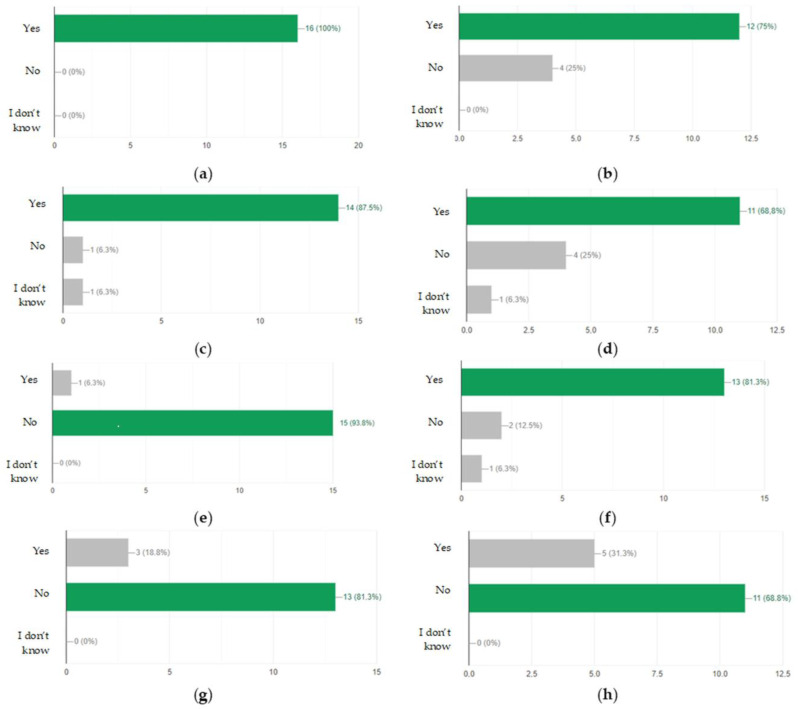
Statistics of results concerning the answers to the questions asked on the selected day of the training: (**a**) Does the place of the warning board display matter? (**b**) Can uncontrolled movement of the belt occur after the power is turned off? (**c**) Should the drive frame be anchored before installing the drums and before positioning the belt? (**d**) Is the positioning of the belt only carried out with the belt take-up unit carriage? (**e**) Can I use a conveyor with a broken emergency cable? (**f**) Could an oil level that is too low contribute to a fire? (**g**) Is the most important thing to do in the event of a fire to notify a supervisor? (**h**) Can machine assemblies be transported by belt?

After the employee sent their answers, they were automatically entered in a previously prepared online file. Statistics on the number of correctly selected answers for each of the questions were kept in another file. The file included such data as the questions with the highest number of wrong answers, the number of people who participated in the survey, or finally, the average and median values for each training day. Statistics of results concerning the answers to the questions is shown in Figure 5.

**Figure 6:**
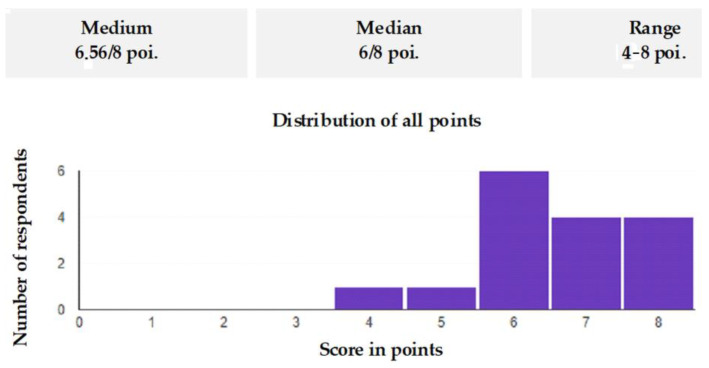
Summary of results from the selected training day.

**Figure 7:**
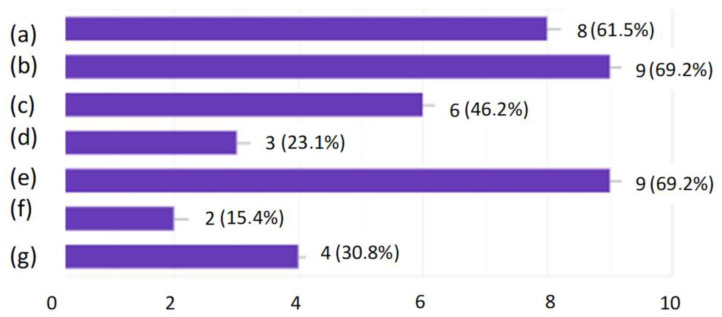
What did the respondents like most about the training? (**a**) I could test my knowledge; (**b**) The quiz showed me the correct answers; (**c**) I liked the form of the training; (**d**) There were only eight questions a day; (**e**) I was able to take the quiz at any time convenient for me; (**f**) The form of the quiz was intuitive; (**g**) Possibility to contact the person responsible for the test.

**Figure 8:**
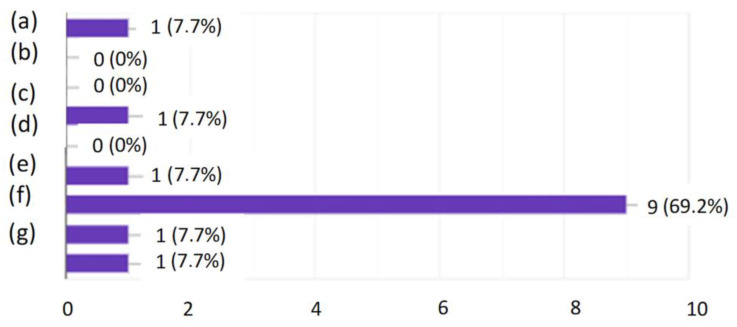
What did the respondents dislike about the training? (**a**) I had to take quizzes every day; (**b**) The questions were difficult; I was getting an SMS reminder about completing them; (**c**) There were as many as eight questions a day; (**d**) Quiz submission time (**e**) The results were at the end of the test, not after each question; (**f**) I liked everything; (**g**) A 16-question option spread over five days would be better.

**Figure 9:**
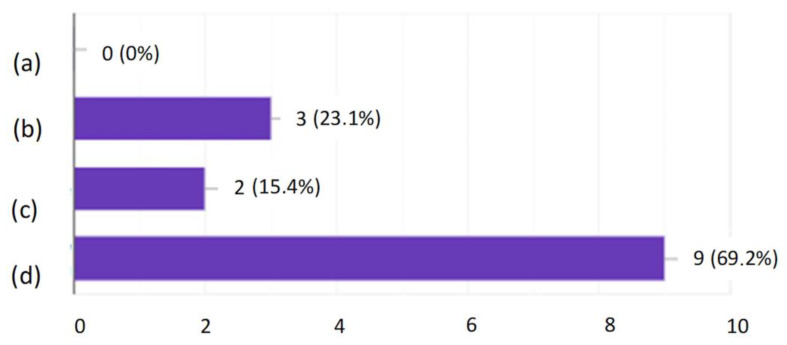
How do you rate the questions asked? (**a**) Difficult; (**b**) Easy; (**c**) Nonstandard; (**d**) They were of varied difficulty, but generally okay.

**Figure 10:**
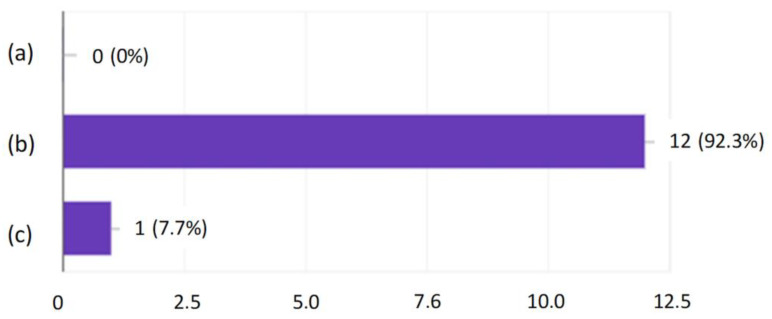
What do you think about the number of questions: (**a**) There were too many of them for one day; (**b**) The number of questions was just right; (**c**) There were not enough of them for one day.

**Figure 11:**
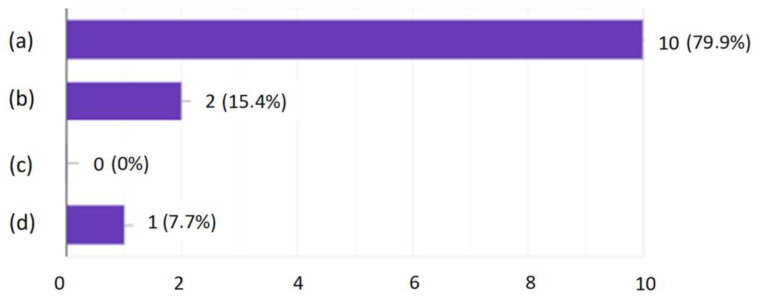
How do you assess the contact with the person responsible for the test: (**a**) Very good; (**b**) Good; (**c**) Difficult; (**d**) Contact was not necessary.

**Figure 12:**
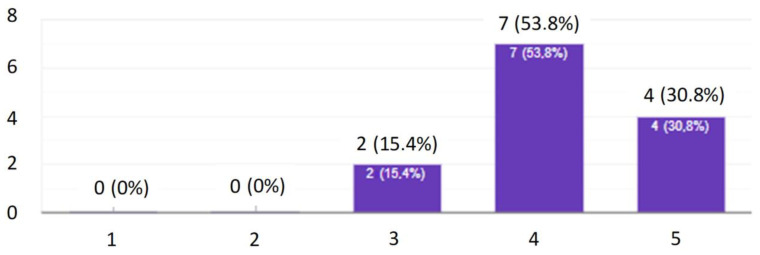
How do you rate the entire study on a scale of 1 to 5?

**Figure 13:**
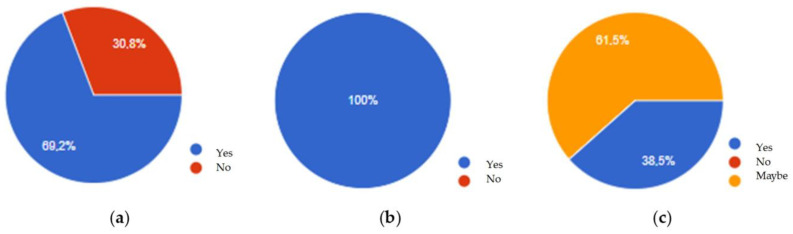
(**a**) Have you gained more knowledge about conveyor belts? (**b**) Was previously acquired knowledge consolidated? (**c**) Would you agree to take part in the study again?

After completing the research, i.e., after conducting ten quizzes, all the results were collected and summarized (Figure 6, Figure 7, Figure 8, Figure 9, Figure 10, Figure 11, Figure 12 and Figure 13). It turned out that despite the fact that the employees answered most of the questions well, it never happened that one of them gained the maximum number of points from each quiz. It follows that each employee has certain areas of knowledge that could use some revision.

In order to check how the employees themselves perceived this form of training, a questionnaire survey was conducted. The research shows that the employees liked the general form of the training. As the strongest points of this method, the respondents indicated the possibility of taking the quiz at any time of the day they choose. The quiz showed them the correct answers after its completion so they knew where they had made a mistake. The success of this method of training is proven by the fact that each of the employees participating in the survey indicated that they had solidified their knowledge. As many as 69.2% of people indicated that they feel that they now have more knowledge about conveyor belts used in a hard coal mine than they had before the training. Based on the entire analysis, it was found that the developed form of active training could be accepted by employees and contribute to an increase in the level of their knowledge and thus to an increase in the level of safety.

The method consists of conducting active training daily for a fixed period of time (for example, two weeks every 6 months). The training can be carried out through short questionnaires that will verify employees’ knowledge on various areas of their activities in order to maintain a high level of work safety. The method places emphasis on knowledge verification, but also systematic reminding (the employee is able to see where they have made mistakes and, when finished, see the correct answers) of occupational health and safety rules.

Using this form of active training, employers can not only increase the knowledge of their employees but also solidify the knowledge they already have. As a result, they can also reduce their work routine, which is one of the most common causes of accidents. Due to the fact that employees would take a short quiz before starting their work, they would consolidate basic safety rules that would remind them of the possible danger. Seasonal training (for example, two weeks every 6 months) would consolidate the knowledge of the trainees without requiring additional routine.

## 5. Conclusions

The development of an active training course for employees operating conveyor belts was a multi-stage task. It consisted mainly of developing four modules with properly formulated questions. On the basis of these modules, the online course was finally created. When analyzing the results of paper tests, which were constructed in a way that makes it possible to check how a given form of questions is perceived by employees, it was noticed that the professional experience and seniority of employees does not determine their level of knowledge, and some employees answered the questions with a correctness rate of only about 50%. The best results (average 80%) were obtained by employees answering questions about the repair and maintenance of the conveyor belt drive unit. However, it should be taken into account that the tests from a given module were performed by employees with various levels of professional experience. The lowest average result was 60%, and it concerned answers to questions related to the operation of the conveyor belt drive unit. It was also in this module that the lowest result of all modules, amounting to 45%, was recorded, and this concerned people with approximately nine years of work experience. In this case, the number of respondents was the highest and amounted to eight people, so the average result is the most representative. In the remaining modules, the average employee result was 66% and 69%, respectively, for assembly and disassembly of the conveyor drive unit and for emergency conditions. The results, which proved to be not very high, confirmed the usefulness of introducing additional training in workplaces.

The online research, based on the questions contained in the modules, was well received by the respondents. Most of them, 84.6%, assessed the entire study as good or very good. Many of them appreciated the possibility of acquiring new knowledge or consolidating subject knowledge related to their activities, thanks to the fact that they were presented with the correct answer to the question asked. Employees also appreciated the possibility of taking the test at any time of the day they chose.

The development of an active training course for employees working with the conveyor belt presented in the paper is the first proposal of this type that has been implemented in the Polish Mining Group. The presented solution is aimed at actively maintaining the employee’s knowledge in the domain of work health and safety. Employees would undergo periodic training every year or, depending on the position, every two years. The developed form of training aims to maintain a high level of knowledge. Reminding employees of a few safety rules at work based on the developed form may result in improved safety at work.

## Figures and Tables

**Table 1:** Questions from the selected training day with correct answers.

Question Number	Question	Answer
1	Is it possible to start the conveyor despite the damaged sound signal for maintenance or repair?	NO
2	Can defective pulleys be a direct cause of belt ignition?	YES
3	Can you use the conveyor to transport yourself to another location without the consent of a supervisor or dispatcher?	NO
4	Is it permissible to switch the drive into reverse while the conveyor is moving?	NO
5	Can the arrangement of the trestles affect the belt guidance?	YES
6	Is it necessary to loosen the belt before replacing the drive drum?	YES
7	Is the fastening of the drive the first thing to do as soon as the straight line on which the conveyor is to be placed has been laid out?	YES
8	Could drum seal failure be the cause of excessive heating?	YES

## Data Availability

Not Applicable.
